# Can Post-Operative Posterior Reversible Encephalopathy Syndrome (PRES) Be Considered an Insidious Rare Surgical Complication?

**DOI:** 10.3390/brainsci13050706

**Published:** 2023-04-23

**Authors:** Alessandro Frati, Daniele Armocida, Fulvio Tartara, Fabio Cofano, Sergio Corvino, Sergio Paolini, Antonio Santoro, Diego Garbossa

**Affiliations:** 1 Istituto di Ricovero e Cura a Carattere Scientifico (I.R.C.C.S), Neuromed, Via Atinense 18, 86077 Pozzilli, Italysrgpaolini@gmail.com (S.P.);; 2 Human Neurosciences Department, Neurosurgery Division “Sapienza” University, AOU Policlinico Umberto I, 00161 Rome, Italy; 3 Headache Science and Neurorehabilitation Center, IRCCS Mondino Foundation, Department of Brain and Behavioral Sciences, University of Pavia, 27100 Pavia, Italy; 4 Neurosurgery, Department of Neuroscience, A.O.U. Città della Salute e della Scienza, University of Turin, 10126 Turin, Italy; 5 Department of Neuroscience, Reproductive and Odontostomatological Sciences, Division of Neurosurgery Università degli Studi di Napoli Federico II, 80131 Naples, Italy; sercorvino@gmail.com

**Keywords:** posterior reversible encephalopathy syndrome, hydrocephalus, surgical complication, surgery

## Abstract

*Introduction:* Posterior reversible encephalopathy syndrome (PRES) is a neurological disorder characterized by neurological symptoms and distinctive neuroimaging findings. There are a few cases reported in the literature in which PRES can occur after surgery, and there is no clear direct relationship between a procedure and its debut. *Methods:* We performed a review of the literature by analyzing all reported cases of PRES syndrome which debuted after a surgical procedure with the aim of identifying the clinical features, the timing of the symptoms’ onset and the therapy of patients suffering from this unusual surgical complication. *Results:* The total number of patients collected was 47, with a mean age of 40.9 years. Postoperative PRES can occur in either pediatric or adult patients (ages 4–82 years). The most frequent form of comorbidity reported was cardiovascular disease (fourteen patients, 29.78%). Sixteen patients (36%) had no relevant risk factors or comorbidities at the time of the surgical procedure. The types of surgery most correlated were cranial neuro and maxillofacial surgery (twenty-one patients, 44.68%) followed by transplant surgery (eight patients, 17%). The time of onset of PRES after surgery occurred within the first 3 weeks (mean time of onset 4.7 days), and when rapidly treated with antihypertensive and antiepileptic drugs appeared to have a reversible and benign course. *Conclusion:* PRES syndrome can be considered a rare complication of procedures and can occur following a wide range of surgeries, especially cranial and transplant surgery. Being able to recognize it in time and treat it ensures a full reversibility of symptoms in most cases.

## 1. Introduction

Posterior reversible encephalopathy syndrome (PRES) is a neurological disorder described by Hinchey in 1996 [1], characterized by a large variety of neurological signs [1,2,3,4] and distinctive neuroimaging findings reflecting vasogenic edema observed predominantly in the posterior regions of the brain [1,5,6]. The onset may be acute or subacute [6], with symptoms developing within a few hours up to several weeks [4,5,7]. Patients may present with signs of encephalopathy [8,9], epileptic seizures [4], and following the frequent involvement of the occipital lobes, visual disturbances such as a deterioration of visual acuity [1]. Less specific neurological symptoms include headache, nausea, vomiting and, depending on the lesions’ location, focal neurological deficits, reported in 5–15% of cases [5]. Both clinical and imaging characteristics are usually reversible [9], even if, on average, about 40% of all patients diagnosed with PRES require intensive care monitoring [8] and treatment due to severe complications such as status epilepticus, cerebral ischemia, intracranial hemorrhage or intracranial hypertension [4,10].

The pathophysiology of PRES is still controversial and unclear. It could be caused by a variety of etiologies, among which uncontrolled hypertension is regarded as the main culprit, observed in about 75% of patients [5,11]. Alterations in the hemodynamic state can lead to PRES, and acute hypertension that overcomes cerebral autoregulation leads to the breakdown of the blood–brain barrier (BBB), cerebral vasodilatation, and transudation of fluid, resulting in brain edema [12]. Because the anterior cerebral circulation is much better supplied with sympathetic innervation than the posterior circulation, the posterior cerebral circulation may be predisposed to a loss of protective vasoconstriction, breakthrough vasodilation and vasogenic edema in the face of acute hypertension [4]. Most patients are markedly hypertensive on presentation; however, some have only mildly increased or even normal blood pressure. There are a few cases reported in the literature in which PRES can occur after surgery; however, there is no clear, direct relationship between a procedure and the onset of this insidious syndrome [13]. No study in the literature identifies PRES as a complication of a surgical procedure, and it is not known whether it may be driven by surgery-related stress or mismanagement of general anesthesia during the procedure [14].

In this review, we collected all cases reported in the literature of PRES arising after surgery with the aim of identifying the causes, clinical features of onset, treatment and management of what could be a dreaded though rare postoperative complication.

## 2. Materials and Methods

We performed a review of the literature by analyzing all reported cases of PRES syndrome debuted after a surgical procedure, with the aim of identifying the clinical features, timing of symptoms’ onset and therapy of patients suffering from this unusual surgical complication.

### Eligibility Criteria

Our target was to identify the type of surgical procedure performed, the time of onset, clinical and time debut, and the clinical prognosis of patients who experienced symptoms attributable to PRES syndrome from the immediate postoperative stage. Therefore, while screening the literature, we adopted the following inclusion criteria:

- Meta-analysis, case series, clinical study or clinical image reporting cases of patients who suffered from PRES syndrome after a surgical procedure.

Conversely, we excluded the following:

- Cases reported without detailed clinical features of patients;
- Cases reported without description of radiological images;
- Papers that report other pathologies (off topic);
- Papers written in languages other than English.

The English literature was systematically investigated using MEDLINE, the NIH Library, Pubmed and Google Scholar. The last search date was 5 May 2022. The following search terms were used: PRES syndrome AND “Post-operative” OR “after surgery”.

Two independent reviewers (D.A. and F.C.) screened each record (title/abstract) and each report retrieved at each stage of screening. Duplicated articles were removed after the first investigation of the libraries.

## 3. Results

**Figure 1:**
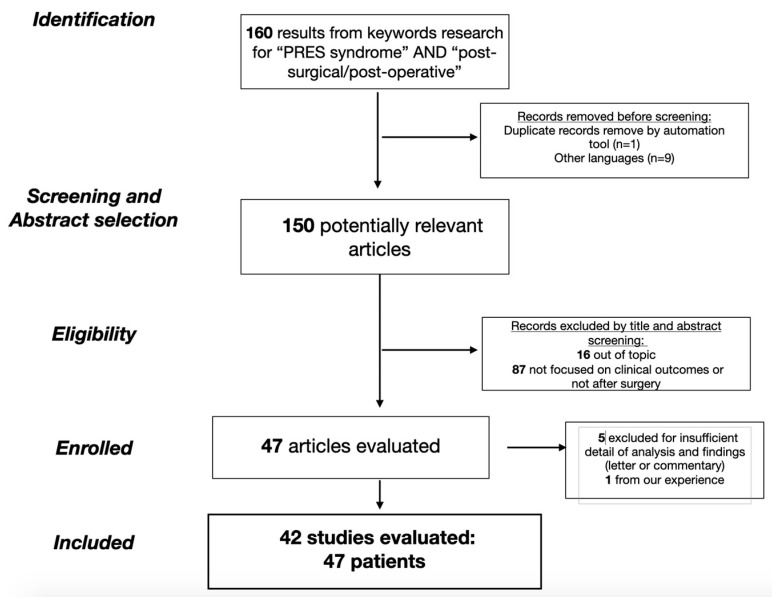
The flow-chart of the study selection with PRISMA criteria.

The search returned a total of 160 papers, including radiological, molecular and clinical studies. In Figure 1, the article selection flow-chart with PRISMA criteria is reported accordingly (Figure 1). To this initial cohort, the aforementioned exclusion criteria were applied, accordingly eliminating a total of 10 papers for duplicated title and language selection. The resulting 150 papers were evaluated by title and abstract: 16 articles were exclude because they were outside the topic (not referring to the PRES syndrome) and 87 articles were excluded because they did not focus on clinical outcome (comprehensive review, book chapters, clinical images) or because they were not in relation to a surgical procedure. Five articles were subsequently excluded after a complete revision of the paper for incomplete data. The list of 42 evaluated articles is given in Table 1.

For each case described, we reported the sex and age of the patient, the presence of comorbidity or risk factors, the type of surgery performed, whether there were peri-operative complications, the time of clinical onset of PRES syndrome, the predominant onset symptomatology, the eventual time of symptom recovery, and the prognostic status (reported as “Good” if there was complete recovery of PRES symptoms, “stable” if there were permanent but controlled changes, “worst” if there was clinical worsening with reduced expectancy or quality of life). From the time of the onset of PRES, all patients experienced symptoms of seizure states and hypertensive states and we reported “seizure” as the main symptom when it was the only onset symptom.

The total number of patients collected for this review was 47, with a mean age of 40.9 years. Postoperative PRES syndrome can occur in either pediatric or adult age patients, with a reported minimum age of 4 years to a maximum of 82 years. There was no clear sex prevalence in the manifestation of the syndrome, with 25 female and 21 male individuals reported (one sex data point was missing in one case report).

The patient comorbidities reported were very varied, including gastroenterological, renal, immune and metabolic diseases. The most frequent form of comorbidity reported was cardiovascular disease (14 patients, 29.78%). Of note, however, a significant percentage of patients had no relevant risk factors or comorbidities at the time of the surgical procedure (16 patients, 36%).

On the other hand, if we consider the type of surgery most correlated with the postoperative onset of PRES syndrome, it shows that the types of surgery most involved are cranial neuro and maxillofacial surgery (twenty-one patients, 44.68% of cases), transplant surgery (eight patients, 17%) and orthopedic and spine surgery (six patients, 12.76%), all three accounting for more than 70% of the surgeries in which this complication may occur.

In the majority of cases (39 patients, 83%), there were no intra-operative complications reported in the selected studies. The time of onset of PRES syndrome after surgery was reported in 45/47 patients, and in most cases (43 patients 91.5%) it occurred within the first 3 weeks (mean time of onset 4.7 days); in two cases, late onset was found at 6 and 10 months (in both cases, after prolonged therapy with immunosuppressive drugs, Tacrolimus). In the cases in which we have reported onset at 1 day, we refer to onset within the first 24 h after cessation of sedation after the procedure.

All reported patients experienced epileptic symptoms, where in 21 cases it was the only manifestation of the syndrome and in 19 cases (40%) there was a loss of acute visual acuity as the initial symptom. We found that even in cases of PRES arising after a surgical procedure, the symptomatology tended to be self-limiting and transient with a full recovery of neurological symptoms occurring in 35 patients (74.5%). In these cases, effective anti-hypertensive and anti-epileptic therapy was introduced at the first manifestation of symptoms.

The time to recovery of Initial neurological status was found to be widely variable, from a minimum of 2 days to a maximum of 18 months, with no apparent correlation with the type of surgery, the presence of hypertension among the risk factors, and the presence of peri-operative complications. A valid statistical analysis could not be performed due to the small number of patients reported. Resumed data are reported in Table 2.

## 4. Discussion

Although numerous case reports and observational studies have been published, the pathophysiology, treatment and prognosis of PRES has remained unclear since its first description [1,7,9,53,54,55]. Currently, there are no diagnostic criteria or guidelines for PRES, and clinical and neuroimaging findings are often not specific [5,56,57]. There are two leading theories regarding the pathophysiology of PRES [9]: the first hypothesis, the ‘vasogenic theory’, proposes a rapid increase in arterial blood pressure up to a hypertensive crisis, which has been observed in most patients at PRES onset [5]. According to this hypothesis, the elevation of blood pressure levels above the upper autoregulatory limit leads to cerebral hyperperfusion [58], causing a breakdown of the BBB and secondary vasogenic edema [9,59,60]. The cerebral hemispheres’ posterior areas seem particularly susceptible, supported by clinical and imaging findings [5]. However, this theory does not explain the mechanism in patients with borderline hypertension and normotensives (30% of patients with PRES [61]). The ‘neuropeptide’ theory regarding the cause of PRES is that the syndrome is triggered by endothelial dysfunction caused by circulating endogenous or exogenous toxins [62], causing vasospasm and ischemia and cerebral edema as a consequence of primary endothelial dysfunction, increased vascular permeability and edema formation [9,53]. Arguing for this hypothesis, PRES is frequently observed in patients with (pre)eclampsia [7], sepsis, trauma, or during treatment regimens with immunosuppressive agents [63] or cytotoxic medication [64,65]. Many authors report that a significant number of PRES cases occurred after a surgical procedure. In this research, we found that while the pathology leading up to the procedure, the therapy, and the sedation used during the procedure were hypothesized to be in relation to the onset of PRES, in fact there does not seem to be a direct correlation with one pathology versus another. We have identified cases of postoperative PRES in patients treated for gastrointestinal pathology [27,30,47], gynecologic pathology [12], and even urologic pathology [31,48]. Several anesthesiologic reports [12,27,31,50] on PRES have argued that the major causative factor could be sub-optimal pressure control in hypertensive patients [25,26]. We found that the most frequent form of comorbidity reported was cardiovascular disease, effectively [44], although it is reported in a percentage less than 30%, with the majority of patients being without known risk factors. Although there are various causes of this syndrome, not all affected patients typically have a history of hypertension at the time of presentation [34,44]. Further, perioperative hypertension occurs in 25% of hypertensive patients undergoing surgery without neurological consequences [66]. With regard to the role of immunosuppressive agents, particularly calcineurin inhibitors [21] and tacrolimus [33], reported in most studies as causative, it is noteworthy that only four of our series reported high plasma levels. It is likely that the mentioned risk factors (hypertension and calcineurin inhibitors) are important but insufficient to cause PRES, and they are thought to be associated with other, still unidentified factors that influence endothelial function, BBB, or fluid homeostasis [33].

**Figure 2:**
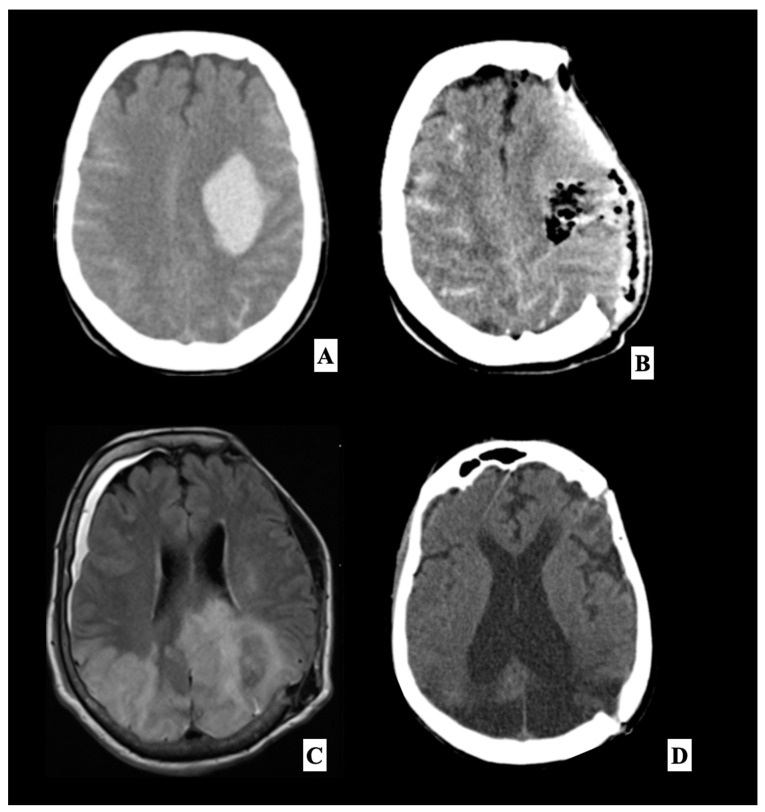
This is a representative case of a 55-year-old woman with no significant previous medical history and essential hypertension, who was transported to our emergency unit after the onset of right hemiplegia with Glasgow Coma Scale (GCS) 6, preceded by a severe headache which occurred two hours earlier. On initial evaluation, blood pressure was elevated at 160/100 mmHg. A head angio computed tomography (**A**) scan performed at our department showed a large left frontotemporal subdural hemorrhage. A left front-temporoparietal decompressive craniectomy was performed to remove the hematoma. Postoperative head CT scan (**B**) showed the surgical results, with complete clot removal with a significant reduction in the midline shift. The patient was therefore transferred to our intensive care unit. During sedation, the patient had poorly controlled hypertensive peaks and the causes of secondary hypertension were excluded (urinary catecholamines analysis, abdominal CT-scan and serum-level hormones). The patient was extubated four days after the craniectomy. The neurological examination documented a slight improvement in the right side motor disorder and a considerable improvement in the consciousness state, with a GCS 10. A brain MRI (**C**) was performed about 3 weeks after surgery, which showed bilateral occipital, parietal and left frontal cortex and subcortical white matter T2/Fluid-attenuated inversion recovery (FLAIR) hyperintensities associated with disruption of the BBB evident after administration of gadolinium, suggestive of PRES. The patient underwent a cranioplasty with her autologous bone flap after three weeks. On follow-up at six weeks, the patient did not present modification of neurological status and reported no recurrence of severe neurological deficits or seizures. She was submitted to an Electroencephalography (EEG) that showed normal, reduced electroencephalographic activity without sign of seizure, and a follow-up head CT scan showed an enlargement of the frontal ventricles (**D**), and the patient was given third ventriculostomy and a ventriculoperitoneal shunt. After one week, the patient showed a general improvement in verbal and motor response.

Each reported case of postoperative PRES was analyzed from the perspective of the pathology of interest, going on to hypothesize, for example, the role of primary damage to the BBB in cases of operated posterior cranial fossa lesions [15,18,46], or immunosuppressive therapy [17,20,21,39,40] in patients undergoing organ transplantation [35,52]. Instead, we believe that in individuals predisposed to have primary endothelial damage, a stressful incident such as surgery of whatever type may result in postoperative PRES in rare cases. In most cases Figure 2, no intra-operative complications were reported in the selected studies. We found that there was a higher percentage for certain types of surgery, such as in the case of neurosurgical pathology [16,24,28,29,32], both cranial and spinal [15,18,19,36,42,49]. In some cases, the risk factor for PRES was sustained by hypertension caused by incomplete postoperative pain control [67]. Adequate pain control may have prevented the development of PRES [34].

So while it is true that it is not, therefore, possible to accurately predict the real risk of developing PRES after a surgical procedure, it should also be understood that since it is a condition with self-limiting symptoms, the operator’s awareness that it is a rare complication can be of profound help in knowing how to recognize it in time and manage it in the most appropriate manner. PRES is a clinical entity that may be unfamiliar to many anesthesiologists and surgeons, but they are often the first clinicians to be confronted with a patient presenting with an acute visual loss after intraoperative hypertension. As such, they should be familiar with this diagnosis. In our opinion, PRES syndrome that occurs after neurosurgical intervention is an infrequent but not rare complication with a relevant risk of not being identified or of being mistaken for a complication of direct tissue or vascular damage after a procedure.

Differentiation from acute cerebral ischemia, and prompt and vigorous treatment of exacerbating factors, such as intermittent hypertension, are important if permanent visual loss is to be avoided [12]. Brain imaging is useful to exclude alternative diagnoses, and usually confirms a PRES diagnosis [5] and suggests any sequelae like hemorrhages [68] or hydrocephalus described in our representative case [56,69]. Computed tomography (CT) scans usually show vasogenic edema with a bi-hemispheric distribution [9]. However, magnetic resonance imaging (MRI) is the most important diagnostic tool; it is more sensitive to displaying hyperintense lesions in T2-weighted or fluid-attenuated inversion recovery (FLAIR) sequences [9]. The typical MRI finding of PRES is remarkable vasogenic edema predominantly involving parieto-occipital regions (sometimes distributed asymmetrically) [70] in both hemispheres [71], with increased apparent diffusion coefficient (ADC) values (useful to determine the prognosis [72,73]) observable on diffusion-weighted imaging (DWI) [70,73,74].

However, cortical involvement has also been described [9,10]. While parieto-occipital distribution occurs in about 70% of all patients, a frontal sulcus or watershed pattern is also frequently seen [9,22,75,76,77]. Lesions in other areas, such as the cerebellum, brain stem, basal ganglia, or spinal cord, are less common [75]. Electroencephalography (EEG) may be necessary for the detection of (non-convulsive) epileptic seizures [76,77], and status epilepticus may also help in the evaluation of encephalopathy [78,79,80,81,82]. Despite the heterogeneity in its etiologies and proposed mechanisms, PRES is a downstream effect characterized by a combination of clinical and radiological features [69].

The treatment of PRES is symptomatic since no specific therapeutic strategy is currently available. Early recognition is crucial, as timely management of its precipitating factor is required to achieve reversibility [4]. No guidelines exist to direct this assessment; therefore, clinical judgment is crucial since PRES diagnosis is not mainly radiological [43]; the clinical context and clinician’s judgment are essential to making the correct diagnosis [5]. Every patient in this series, during or after the onset of neurological symptoms, presented a state of hypertension. Although no randomized control trials have been held to assess the effect of blood pressure management on PRES resolution, the consensus among physicians suggests that the control of hypertensive episodes and normal blood pressure maintenance is an essential component of treatment [5,72,83]. An early indication of PRES could lead to timely [51], appropriate management of this potentially reversible and treatable disorder, resulting in positive clinical outcomes. Anticonvulsive treatment is frequently required. There Is no general recommendation for the use of specific drugs [72], and specific prognostic factors have not been identified. Although PRES-associated clinical signs and symptoms and neuroimaging lesions are reversible in the majority of patients, the prognosis is mainly determined by the underlying pathology [23,38,41,45,84,85] and the following treatment, considering that possible neurological sequelae, in particular, reduced visual acuity and epilepsy, may persist in individual cases. Large, multi-center prospective studies using inclusive diagnostic criteria would be valuable to examine associations with surgery, atypical manifestations and prognosis.

## 5. Conclusions

PRES is a complex and difficult to recognize neurological syndrome probably caused by an intrinsic endothelial defect that may arise following several stressogenic insults including a surgical procedure. Recognizing PRES as a surgical complication allows its rapid diagnosis and management while achieving good clinical outcomes.

## Figures and Tables

**Table 1:** PRES syndrome and study population.

No.	Authors	Year	No. Pts.	Age	Sex	Comorbidity/Risk Factors	Pathology	Complications of Procedure	Time of Onset (Days)	Neurological Symptoms	Time of Symptoms Relapse (Days)	Outcome	Complications or Notes
1	Moriarity JL et al. [15]	2001	1	19	M	None	Posterior Fossa Tumor	Hypo-hypertension	1	Seizure	56	Good	/
2	Triquenot-Bagan et al. [13]	2003	1	55	M	Ischemic cardiopathy	Abdominal aortic aneurysm	No	4	Visual loss, headache	13	Good	Hypertension residual
3	Horbinski C. et al. [16]	2009	1	57	M	Hypertension	Cardiac transplant	No	5	Seizure	/	/	
4	Won SC et al. [17]	2009	2	6	F	None	Neuroblastoma	No	1	Seizure	5 months	Good	
5				11	F	None	Osteosarcoma	No	1	Visual loss, seizure	3	Good	
6	Kim TK et al. [12]	2010	1	44	F	None	Uterin Mioma	Hypertension	1	Visual loss, headache	1	Good	
7	Patel AJ et al. [18]	2010	1	6	M	None	Pylocitic astrocytoma	Hypertension	1	Low cranial nerves injury	14	Good	/
8	Gopalakrishan et al. [19]	2011	1	14	F	None	Hemangioma dorsal	Hemorrhage	1	Seizure	4	Good	/
9	Sanchez-Cuadrado et al. [20]	2011	1	58	M	None	Head-neck tumor (ear)	No	2	Confusion, visual loss	5	Good	
10	Santos MM et al. [21]	2011	2	9	M	Biliar athresy	Liver transplant	No	8	Seizure, hemiparesis	28	Good	/
11				13	F	Alagylle’s syndrome	Liver transplant	No	3	Seizure, hypertension	3 months	Good	/
12	Yi JH. et al.	2011	1	71	F	Cardiomegaly	Spinal lumbar stenosis L4-L5	No	1	Seizure	8	Good	
13	Sadek A-R et al. [22]	2012	1	51	F	Hypertension	SAH aneurysm	No	19	Visual loss, hemiparesis	/	Worse	Coma
14	Avecillas-Chasin JM et al. [23]	2013	1	19	M	None	Posterior Fossa Tumor	No	7	Visual loss, seizure	7	Good	
15	Hansberry DR et al. [24]	2013	1	25	F	None	Chiari malformation	No	6	Hemiparesis, swallowing deficit	2	Stable	Hemiparesis
16	Kuhnt D. et al. [25]	2013	1	63	M	Hypertension	Intracranial Hemangiopericytoma	Hypotension-hypertension	1	Visual loss, ophthalmoplegia	8	Stable	Reduced visual acuity
17	Riaz N. et al. [26]	2013	1	56	M	Smoking	Lung cancer	Bronco-pleural fistula	3	Visual loss, confusion	10	Good	
18	Shah R. et al. [27]	2014	1	62	F	Hypertension, Celiac disease	Peritonitis, colitis	No	3	Visual loss, headache	21	Stable	Reduced visual acuity
19	González Quarante LH et al. [28]	2015	2	4	M	None	Medulloblastoma	No	9	Seizure	1 month	Good	
20				14	M	None	Medulloblastoma	No	2	Seizure	14	Good	
21	Sorour M. et al. [29]	2015	1	57	F	Hypothyroidism	Vestibular schwannoma	No	2	Seizure	13	Good	
22	Stanford FC et al. [30]	2015	1	61	F	Carotid stenosis, hyperlipidemia, obesity	Bariatic surgery	No	21	Visual loss, hemiparesis	1 month	Good	IRC
23	Elkoundi A. et al. [31]	2016	1	67	M	Hypertension, IPB	TURP	No	1	Blindness	2	Stable	Blind
24	Fok A. et al. [32]	2016	1	33	F	Hypertension	Idiopathic intracranial hypertension	No		Seizure, hemiparesis	14	Stable	Reduced visual acuity
25	Giussani A. et al. [33]	2016	2	7	M	None	End-stage renal disease	No	5	Seizure	1 month	Good	Tacrolimus therapy
26				6	M	None	End-stage renal disease	No	10	Seizure, confusion	21	Good	
27	Sato N et al. [34]	2016	1	46	F	None	Leiomioma uterin	No	1	Seizure	49	Good	/
28	Scarpino M et al. [35]	2016	1	69	/	Aortic stenosis	Aortic plasty	No	1	Confusion, visual loss	1 month	Good	/
29	Wakasaki T et al. [36]	2016	1	56	F	Gastritis, stroke	Head-neck tumor	No	4	Visual loss, headache	28	Good	Epilepsy
30	Vakharia K. et al. [37]	2016	1	60	M	Renal cancer	Spinal renal metastases	No	1	Visual loss	2 months	Good	
31	Abusabha Y. et al. [38]	2017	1	52	M	Hypertension	Posterior Fossa Tumor	No	1	Coma	21	Stable	
32	Ban SP et al. [39]	2017	1	52	M	Dilatative cardiomyopathy	Heart transplant	No	8	Seizure	3 months	Good	/
33	Davi CB et. al. [40]	2017	1	48	F	Polycystic kidney	Kidney transplant	IRA	10	Lethargy, emianopsy	2	Good	/
34	Hernandez-Duran S. et al. [41]	2017	1	44	F	Hypertension, obesity, DM II	Idiopathic intracranial hypertension	No	1	Coma	42	Good	
35	Ibrahim TF et al. [42]	2017	1	67	M	Hypertension, kidney failure	Degenerative scoliosis	IRA	6	Visual loss	2	Good	/
36	Magsi et al. [43]	2017	1	62	F	Hyperlipidemia, colitis	Intraparenchymal spontaneous hemorrhage	No	4	Visual loss	3 months	Good	/
37	Villelli NW et al. [44]	2017	1	59	F	None	Pituitary adenoma	No	1	Visual loss, Headache	18 months	Stable	Residual visual deficit
38	Delgado-Lopez et al. [45]	2018	1	82	F	Hypertension	Spinal lumbar stenosis L4-L5	No	1	Seizure	12	Stable	Residual hemiparesis
39	Khatri D. et al. [46]	2018	1	23	F	None	Vestibular schwannoma	No	6 months	Seizure	14	Stable	Residual epilepsy
40	Kerkeni Y. et al. [47]	2018	1	13	F	Duplication uterine cyst	Surgical asportation	No	7	Seizure	9 months	Good	
41	Magray MA et al. [48]	2018	1	8	M	Meningocele	Neurogenic bladder	No	12	Visual loss	29	Good	
42	Zimering J. et al. [14]	2018	1	68	F	DM II, AR	C5-C6 subluxation	No		Seizure	14	Good	
43	Oxford BG et al. [49]	2019	1	69	M	Hypertension, hyperlipidemia	Rathke Cistis	No	2	Visual loss	6 months	Good	Hypertension residual
44	Liu J-F et al. [50]	2020	1	40	F	Hepatitis	Hepatic transplant	No	10 months	Seizure	7	Good	
45	Rastogi A et al. [51]	2020	1	12	M	Thalassemia	Splenectomy	No	2	Visual loss, Headache	7	Good	/
46	Wong M. et al. [52]	2020	1	51	F	None	Intracranial suprasellar mass	No	4	Visual loss	7	Good	
47	Our cases	2020	1	55	F	Hypertension	Intraparenchymal spontaneous hemorrhage	No	21	Seizure	42	Stable	Hydrocephalus 4 weeks after surgery

Abbreviations: IRA: Acute renal insufficiency, IPB: Prostatic benign hypertrophy, DM II: Diabetes Mellitus type II, AR: Rheumatoid arthritis.

**Table 2:** Study analysis, patient’s analysis.

**No. Patients**	47	
**Age**	Mean: 40.9	Min: 4 Max: 82
**Sex**	F: 25 M: 21	
**Comorbidity and risk factors**	Cardiopathy: 14 pts—29.78%	
	No risk factors or comorbidity: 16 pts—34%	
**Surgical Procedure**	Cranial surgery: 21 pts	44.68%
	Organ transplant: 8 pts	17%
	Orthopedic-spine surgery: 6 pts	12.77%
	Abdominal/general surgery: 4 pts	8.51%
	Gynecology: 3 pts	6.4%
	Vascular: 2 pts	4.26%
	Urology: 2 pts	4.26%
	Thoracic surgery: 1 pt	2.13%
**Eventual complications of procedure**	None: 39 pts	
	Blood pressure instability: 4 pts	
	IRA: 2 pts	
	Bronco-pleural fistula: 1 pt	
	Hemorrhage: 1 pt	
**Time of onset of PRES**	Mean: 4.1 days	Min: 1 day Max: 10 months
**Neurological Symptoms**	Visual loss/reduced acuity: 19 pts	
	Seizure: 21 pts	
	Hemiparesis: 5 pts	
	Dizziness/confusion: 5 pts	
	Cranial nerves injury: 2 pts	
	Coma: 2 pts	
**Eventual time of symptoms relapse**	Mean: 45.2 days	Min: 1 day Max: 18 months
**Prognosis**	Good: 35 pts (74.5%)	
	Stable: 10 pts (21.3%)	
	Worst: 1 pt (0.02%)	

## Data Availability

Not applicable.

## References

[1] Hinchey J., Chaves C., Appignani B., Breen J., Pao L., Wang A., Pessin M.S., Lamy C., Mas J.L., Caplan L.R. (1996). A reversible posterior leukoencephalopathy syndrome. N. Engl. J. Med..

[2] Saad A.F., Chaudhari R., Wintermark M. (2019). Imaging of Atypical and Complicated Posterior Reversible Encephalopathy Syndrome. Front. Neurol..

[3] Bing F., M’biene S., Gay S. (2020). Brainstem Posterior Reversible Encephalopathy Syndrome with Spinal Cord Involvement (PRES-SCI). Rev. Neurol..

[4] Lee V.H., Wijdicks E.F., Manno E.M., Rabinstein A.A. (2008). Clinical Spectrum of Reversible Posterior Leukoencephalopathy Syndrome. Arch. Neurol..

[5] Fugate J.E., Rabinstein A.A. (2015). Posterior reversible encephalopathy syndrome: Clinical and radiological manifestations, pathophysiology, and outstanding questions. Lancet Neurol..

[6] Brubaker L.M., Smith J.K., Lee Y.Z., Lin W., Castillo M. (2005). Hemodynamic and Permeability Changes in Posterior Reversible Encephalopathy Syndrome Measured by Dynamic Susceptibility Perfusion-Weighted MR Imaging. Am. J. Neuroradiol..

[7] Liman T.G., Bohner G., Heuschmann P.U., Endres M., Siebert E. (2011). The clinical and radiological spectrum of posterior reversible encephalopathy syndrome: The retrospective Berlin PRES study. J. Neurol..

[8] Hinduja A. (2019). Posterior Reversible Encephalopathy Syndrome: Clinical Features and Outcome. Front. Neurol..

[9] Bartynski W.S., Boardman J.F. (2007). Distinct Imaging Patterns and Lesion Distribution in Posterior Reversible Encephalopathy Syndrome. AJNR Am. J. Neuroradiol..

[10] Kummer S., Schaper J., Mayatepek E., Tibussek D. (2010). Posterior Reversible Encephalopathy Syndrome in Early Infancy. Klin. Padiatr..

[11] Rykken J.B., McKinney A.M. (2014). Posterior reversible encephalopathy syndrome. Semin. Ultrasound. CT MR.

[12] Kim T.K., Yoon J.U., Park S.-C., Lee H.J., Kim W.S., Yoon J.Y. (2010). Postoperative blindness associated with posterior reversible encephalopathy syndrome: A case report. J. Anesth..

[13] Triquenot-Bagan A., Gerardin E., Guegan-Massardier E., Onnient Y., Leroy F., Mihout B. (2003). Postoperative Reversible Posterior Leukoencephalopathy Syndrome. Cerebrovasc. Dis..

[14] Zimering J.H., Mesfin A. (2016). Posterior reversible encephalopathy syndrome following elevated mean arterial pressures for cervical spinal cord injury. J. Spinal. Cord. Med..

[15] Moriarity J.L., Lim M., Storm P.B., Beauchamp N.J., Olivi A. (2001). Reversible Posterior Leukoencephalopathy Occurring during Resection of a Posterior Fossa Tumor: Case Report and Review of the Literature. Neurosurgery.

[16] Horbinski C., Bartynski W.S., Carson-Walter E., Hamilton R.L., Tan H.P., Cheng S. (2008). Reversible encephalopathy after cardiac transplantation: Histologic evidence of endothelial activation, T-cell specific trafficking, and vascular endothelial growth factor expression. Am. J. Neuroradiol..

[17] Won S.C., Kwon S.Y., Han J.W., Choi S.Y., Lyu C.J. (2009). Posterior Reversible Encephalopathy Syndrome in Childhood With Hematologic/Oncologic Diseases. Pediatr. Hematol. Oncol..

[18] Patel A.J., Fox B.D., Fulkerson D.H., Yallampalli S., Illner A., Whitehead W.E., Curry D.J., Luerssen T.G., Jea A. (2010). Posterior reversible encephalopathy syndrome during posterior fossa tumor resection in a child. J. Neurosurg. Pediatr..

[19] Gopalakrishnan C.V., Vikas V., Nair S. (2011). Posterior Reversible Encephalopathy Syndrome in a Case of Postoperative Spinal Extradural Haematoma: Case Report and Review of Literature. Asian Spine J..

[20] Sanchez-Cuadrado I., Lassaletta L., Royo A., Cerdeño V., Roda J.M., Gavilán J. (2011). Reversible Posterior Leukoencephalopathy Syndrome after Lateral Skull Base Surgery. Otol. Neurotol..

[21] Santos M.M., Tannuri A.C., Gibelli N.E., Ayoub A.A., Maksoud-Filho J.G., Andrade W.C., Velhote M.C., Silva M.M., Pinho M.L., Miyatani H.T. (2010). Posterior reversible encephalopathy syndrome after liver transplantation in children: A rare complication related to calcineurin inhibitor effects. Pediatr. Transplant..

[22] Sadek A.R., Waters R.J., Sparrow O.C. (2012). Posterior reversible encephalopathy syndrome: A case following reversible cerebral vasoconstriction syndrome masquerading as subarachnoid haemorrhage. Acta Neurochir..

[23] Avecillas-Chasín J.M., Gómez G., Jorquera M., Alvarado L.R., Barcia J.A. (2013). Delayed posterior reversible encephalopathy syndrome (PRES) after posterior fossa surgery. Acta Neurochir..

[24] Hansberry D.R., Agarwal N., Tomei K.L., Goldstein I.M. (2013). Posterior reversible encephalopathy syndrome in a patient with a Chiari I malformation. Surg. Neurol. Int..

[25] Kuhnt D., Becker A., Benes L., Nimsky C. (2013). Reversible Cortical Blindness and Internuclear Ophthalmoplegia after Neurosurgical Operation: Case Report and Review of the Literature. J. Neurol. Surg. Part A Central Eur. Neurosurg..

[26] Riaz N., Behnia M.M., Catalano P.W., Davis J. (2013). A Patient with Moderate Post-Operative Hypertension Presenting with Posterior Reversible Encephalopathy Syndrome: A Case Report. Tanaffos.

[27] Shah R., Kubisz-Pudelko A., Reid J. (2014). Posterior reversible encephalopathy syndrome following an inadvertent dural puncture during an emergency laparotomy for ischemic colitis—A case report. Local Reg. Anesth..

[28] González Quarante L.H., Mena-Bernal J.H., Martín B.P., Ramírez Carrasco M., Muñoz Casado M.J., Martínez de Aragón A., de las Heras R.S. (2016). Posterior reversible encephalopathy syndrome (PRES): A rare condition after resection of posterior fossa tumors: Two new cases and review of the literature. Childs Nerv. Syst..

[29] Sorour M., Sayama C., Couldwell W.T. (2015). Posterior Reversible Encephalopathy Syndrome after Surgical Resection of a Giant Vestibular Schwannoma: Case Report and Literature Review. J. Neurol. Surg. A Cent. Eur. Neurosurg..

[30] Stanford F.C., Pratt J.S., Meireles O.R., Bredella M.A. (2015). Posterior reversible encephalopathy syndrome (PRES) after bariatric surgery—A potential consequence associated with rapid withdrawal of antihypertensive medications. BMJ Case Rep..

[31] Elkoundi A., Bensghir M., Meziane M., Haimeur C. (2016). Perioperative visual loss following transurethral resection surgery: Not always a transurethral resection syndrome. Can. J. Anaesth..

[32] Fok A., Chandra R., Gutman M., Ligtermoet M., Seneviratne U., Kempster P. (2016). Posterior Reversible Encephalopathy Syndrome and Subarachnoid Hemorrhage after Lumboperitoneal Shunt for Fulminant Idiopathic Intracranial Hypertension. J. Neuroophthalmol..

[33] Giussani A., Ardissino G., Belingheri M., Dilena R., Raiteri M., Pasciucco A., Colico C., Beretta C. (2015). Posterior reversible encephalopathy syndrome after kidney transplantation in pediatric recipients: Two cases. Pediatr. Transplant..

[34] Sato N., Machida H., Kodaka M., Nishiyama K., Komori M. (2016). Perioperative posterior reversible encephalopathy syndrome in a patient with no history of hypertension: A case report. JA Clin. Rep..

[35] Scarpino M., Olivo G., Quilghini P., Lanzo G., Moretti M., Carrai R., Fontanari P., Amantini A., Grippo A. (2016). Cortical Blindness After Cardiac Surgery: Just an Ischemic Mechanism?. J. Cardiothorac. Vasc. Anesth..

[36] Wakasaki T., Gotoh S., Tomonobe E., Mihara T., Fukushima J. (2016). Posterior Reversible Encephalopathy Syndrome during Combined Modality Therapy for Head and Neck Squamous Cell Carcinoma. Ann. Otol. Rhinol. Laryngol..

[37] Vakharia K., Siasios I., Dimopoulos V.G., Pollina J. (2016). Posterior Reversible Encephalopathy Syndrome Resolving Within 48 Hours in a Normotensive Patient Who Underwent Thoracic Spine Surgery. J. Clin. Med. Res..

[38] Abusabha Y., Petridis A.K., Kraus B., Kamp M.A., Steiger H.-J., Beseoglu K. (2017). Life-threatening posterior reversible encephalopathy syndrome in the cerebellum treated by posterior fossa decompression. Acta Neurochir..

[39] Ban S.P., Hwang G., Kim C.H., Kwon O.K. (2017). Reversible cerebral vasoconstriction syndrome combined with posterior reversible encephalopathy syndrome after heart transplantation. J. Clin. Neurosci..

[40] Davi C.B., Moraes B.P., Lichtenfels B.F., Castro Filho J.B.S., Portal M.M., Montenegro R.M., Manfro R.C. (2018). Posterior reversible leukoencephalopathy syndrome (PRES) after kidney transplantation: A case report. Braz. J. Nephrol..

[41] Hernández-Durán S., Barrantes-Freer A., Rohde V., Von Der Brelie C. (2017). Posterior reversible encephalopathy syndrome presenting in the anterior circulation with malignant intracranial hypertension requiring surgical decompression: A case report and literature review. Acta Neurochir..

[42] Ibrahim T.F., Sweis R.T., Nockels R.P. (2017). Reversible postoperative blindness caused by bilateral status epilepticus amauroticus following thoracolumbar deformity correction: Case report. J. Neurosurg. Spine.

[43] Magsi S., Zafar A. (2017). Malignant Posterior Reversible Encephalopathy Syndrome—An Exacting Challenge for Neurocritical Care Physicians. Neurohospitalist.

[44] Villelli N.W., Prevedello D.M., Ikeda D.S., Montaser A.S., Otto B.A., Carrau R.L. (2017). Posterior Reversible Encephalopathy Syndrome Causing Vision Loss Following Endoscopic Endonasal Resection of Pituitary Adenoma: A Case Report. World Neurosurg..

[45] Delgado-López P.D., Garcés-Pérez G., García-Carrasco J., Alonso-García E., Gómez-Menéndez A.I., Martín-Alonso J. (2018). Posterior Reversible Encephalopathy Syndrome with Status Epilepticus Following Surgery for Lumbar Stenosis and Spondylolisthesis: Case report. World Neurosurg..

[46] Khatri D., Bhaisora K.S., Parab A., Srivastava A.K., Das K.K. (2018). Unusual Delayed Presentation of Posterior Reversible Encephalopathy Syndrome Following Vestibular Schwannoma Surgery: A Rare Neurologic Emergency. World Neurosurg..

[47] Kerkeni Y., Louati H., Hamzaoui M. (2018). Intestinal duplication revealed by posterior reversible encephalopathy syndrome. Korean J. Pediatr..

[48] Magray M.A., Mufti G.N., Bhat N.A., Baba A.A., Buch M.H., Hasan F.U., Banday S.B. (2018). Posterior Reversible Encephalopathy Syndrome after Augmentation Cystoplasty in a Child with Neurogenic Bladder. J. Indian Assoc. Pediatr. Surg..

[49] Oxford B.G., Khattar N.K., Adams S.W., Schaber A.S., Williams B.J. (2019). Posterior reversible encephalopathy syndrome with lumbar drainage and surgery: Coincidence or correlation? A case report. BMC Neurol..

[50] Liu J.F., Shen T., Zhang Y.T. (2020). Posterior reversible encephalopathy syndrome and heart failure tacrolimus-induced after liver transplantation: A case report. World J. Clin. Cases.

[51] Rastogi A., Kaur J., Hyder R., Bhaskar B., Upadhyaya V., Rai A.S. (2020). A case of post-operative posterior reversible encephalopathy syndrome in children: A preventable neurological catastrophe. Indian J. Anaesth..

[52] Wong M., Rajendran S., Bindiganavile S.H., Bhat N., Lee A.G., Baskin D.S. (2020). Posterior Reversible Encephalopathy Syndrome After Transsphenoidal Resection of Pituitary Macroadenoma. World Neurosurg..

[53] Fugate J.E., Claassen D.O., Cloft H.J., Kallmes D.F., Kozak O.S., Rabinstein A.A. (2010). Posterior Reversible Encephalopathy Syndrome: Associated Clinical and Radiologic Findings. Mayo Clin. Proc..

[54] Granata G., Greco A., Iannella G., Granata M., Manno A., Savastano E., Magliulo G. (2015). Posterior reversible encephalopathy syndrome—Insight into pathogenesis, clinical variants and treatment approaches. Autoimmun. Rev..

[55] Yamamoto H., Natsume J., Kidokoro H., Ishihara N. (2015). Clinical and neuroimaging find- ings in children with posterior reversible encephalopathy syn-drome. Eur. J. Paediatr. Neurol..

[56] Sharma A., Whitesell R.T., Moran K.J. (2009). Imaging pattern of intracranial hemorrhage in the setting of posterior reversible encephalopathy syndrome. Neuroradiology.

[57] Tetsuka S., Ogawa T. (2019). Posterior reversible encephalopathy syndrome: A review with emphasis on neuroimaging characteristics. J. Neurol. Sci..

[58] Strandgaard S., Olesen J., Skinhoj E., Lassen N.A. (1973). Autoregulation of brain circulation in severe arterial hypertension. Br. Med. J..

[59] Chen Z., Shen G.Q., Lerner A., Gao B. (2017). Immune system activation in the pathogenesis of posterior reversible encephalopathy syndrome. Brain Res. Bull..

[60] Lassen N. (1971). A Regulation of cerebral circulation. Acta Anaesthesiol. Scand. Suppl..

[61] Feske S.K. (2011). Posterior reversible encephalopathy syndrome: A review. Semin. Neurol..

[62] Bartynski W.S. (2008). Posterior reversible encephalopathy syndrome, part 2: Controversies surrounding pathophysiology of vasogenic edema. AJNR Am. J. Neuroradiol..

[63] Gauiran D.V.T., Lladoc-Natividad T.E.B., Rocha I.I., Manapat-Reyes B.H. (2018). Seizure and acute vision loss in a Fillipino lupus patient: A case of posterior reversible encephalopathy syndrome with intraparenchymal hemorrhage. Case Rep. Med..

[64] Mayama M., Uno K., Tano S., Yoshihara M., Ukai M., Kishigami Y., Ito Y., Oguchi H. (2016). Incidence of posterior reversible encephalopathy syndrome in eclamptic and patients with preeclampsia with neurologic symptoms. Am. J. Obstet. Gynecol..

[65] Marra A., Vargas M., Striano P., Del Guercio L., Buonanno P., Servillo G. (2014). Posterior reversible encephalopathy syndrome: The endothelial hypotheses. Med. Hypotheses.

[66] Goldman L., Caldera D.L., Nussbaum S.R., Southwick F.S., Krogstad D., Murray B., Burke D.S., O’Malley T.A., Goroll A.H., Caplan C.H. (1977). Multifactorial Index of Cardiac Risk in Noncardiac Surgical Procedures. N. Engl. J. Med..

[67] Yi J.H., Ha S.H., Kim Y.K., Choi E.M. (2011). Posterior reversible encephalopathy syndrome in an untreated hypertensive patient after spinal surgery under general anesthesia—A case report. Korean J. Anesthesiol..

[68] Zhang Y.X., Zheng Y., Zhang B.J., Zhang Y., Ding M.P., Zhang B.R. (2016). Variant type of posterior reversible encephalopathy syndrome with diffuse cerebral white matter and brainstem involvement associated with intracranial hemorrhage. J. Stroke Cerebrovasc. Dis..

[69] Yamagami K., Maeda Y., Iihara K. (2019). Variant Type of Posterior Reversible Encephalopathy Syndrome Associated with Deep Brain Hemorrhage: Case Report and Review of the Literature. World Neurosurg..

[70] McKinney A.M., Short J., Truwit C.L., McKinney Z.J., Lozak O.S., SantaCruz K.S., Teksam M. (2007). Posterior Reversible Encephalopathy Syndrome: Incidence of Atypical Regions of Involvement and Imaging Findings. Am. J. Roentgenol..

[71] MacKenzie E.T., Strandgaard S., Graham D.I., Jones J.V., Harper A.M., Farrar J.K. (1976). Effects of acutely induced hypertension in cats on pial arteriolar caliber, local cerebral blood flow, and the blood-brain barrier. Circ. Res..

[72] Lamy C., Oppenheim C., Mas J.L. (2014). Posterior reversible encephalopathy syndrome. Handb. Clin. Neurol..

[73] Karia S.J., Rykken J.B., McKinney Z.J., Zhang L., McKinney A.M. (2016). Utility and significance of gadolinium-based contrast enhancement in posterior reversible encephalopathy syndrome. AJNR Am. J. Neuroradiol..

[74] Armocida D., Marzetti F., Pesce A., Caporlingua A., D’Angelo L., Santoro A. (2019). Purely Meningeal Intracranial Relapse of Melanoma Brain Metastases After Surgical Resection and Immunotherapy as a Unique Disease Progression Pattern: Our Experience and Review of the Literature. World Neurosurg..

[75] Ou S., Xia L., Wang L., Xia L., Zhou Q., Pan S. (2018). Posterior Reversible Encephalopathy Syndrome With Isolated Involving Infratentorial Structures. Front. Neurol..

[76] Kastrup O., Gerwig M., Frings M., Diener H.C. (2012). Posterior reversible encephalopathy syndrome (PRES): Electroencephalo- graphic findings and seizure patterns. J. Neurol..

[77] Armocida D., Pesce A., Frati A., Miscusi M., Paglia F., Raco A. (2019). Pneumoventricle of Unknown Origin: A Personal Experience and Literature Review of a Clinical Enigma. World Neurosurg..

[78] Armocida D., Arcidiacono U.A., Palmieri M., Pesce A., Cofano F., Picotti V., Salvati M., D’andrea G., Garbossa D., Santoro A. (2022). Intracranial Meningioma in Elderly Patients. Retrospective Multicentric Risk and Surgical Factors Study of Morbidity and Mortality. Diagnostics.

[79] Chardain A., Mesnage V., Alamowitch S., Bourdain F. (2016). Posterior reversible encephalopathy syndrome (PRES) and hypomagnesemia: A frequent association?. Rev. Neurol..

[80] Gao B., Yu B.X., Li R.S., Zhang G., Xie H.Z., Liu F.L., Lv C. (2015). Cytotoxic edema in posterior reversible encephalopathy syn- drome: Correlation of MRI features with serum albumin levels. AJNR Am. J. Neuroradiol..

[81] Pirker A., Kramer L., Voller B., Loader B., Auff E., Prayer D. (2011). Type of edema in posterior reversible encephalopathy syndrome depends on serum albumin levels: An MR imaging study in 28 patients. AJNR Am. J. Neuroradiol..

[82] D’angelo L., Armocida D., Sampirisi L., Paglia F., Berra L.V., Santoro A. (2020). Role of endoscopic surgical biopsy in diagnoses of intraventricular/periventricular tumors: Review of literature including a monocentric case series. Acta Neurol. Belg..

[83] James P.A., Oparil S., Carter B.L., Cushman W.C., Dennison-Himmelfarb C. (2014). 2014 evidence-based 005 guideline for the management of high blood pressure in adults: Report from the panel members appointed to the Eighth Joint National Committee (JNC 8). JAMA.

[84] Aranas R.M., Prabhakaran S., Lee V.H. (2009). Posterior Reversible Encephalopathy Syndrome Associated with Hemorrhage. Neurocritical. Care.

[85] Yamashita T., Hiramatsu H., Sakai N., Namba H. (2012). Cerebral Hemorrhage Due to Posterior Reversible Encephalopathy Syndrome Associated With Autonomic Dysreflexia in a Spinal Cord Injury Patient. Neurol. Medico-Chir..

